# Exploring the putative role of PRDM1 and PRDM2 transcripts as mediators of T lymphocyte activation

**DOI:** 10.1186/s12967-023-04066-x

**Published:** 2023-03-24

**Authors:** Erika Di Zazzo, Monica Rienzo, Amelia Casamassimi, Caterina De Rosa, Nicola Medici, Patrizia Gazzerro, Maurizio Bifulco, Ciro Abbondanza

**Affiliations:** 1 Department of Medicine and Health Sciences “V. Tiberio”, University of Molise, 86100 Campobasso, Italy; 2 Department of Environmental, Biological, and Pharmaceutical Sciences and Technologies, University of Campania “Luigi Vanvitelli”, 81100 Caserta, Italy; 3 Department of Precision Medicine, University of Campania “Luigi Vanvitelli”, 80138 Naples, Italy; 4 Department of Pharmacy, University of Salerno, 84084 Salerno, Fisciano (SA) Italy; 5 Department of Molecular Medicine and Medical Biotechnologies, University of Naples “Federico II”, 80131 Naples, Italy

**Keywords:** PRDM1/BLIMP1, PRDM2/RIZ, T lymphocyte activation, T lymphocyte commitment, Transcription factors, Transcription regulation

## Abstract

**Background:**

T cell activation and programming from their naïve/resting state, characterized by widespread modifications in chromatin accessibility triggering extensive changes in transcriptional programs, is orchestrated by several cytokines and transcription regulators. *PRDM1* and *PRDM2* encode for proteins with PR/SET and zinc finger domains that control several biological processes, including cell differentiation, through epigenetic regulation of gene expression. Different transcripts leading to main protein isoforms with (PR +) or without (PR-) the PR/SET domain have been described. Although many studies have established the critical PRDM1 role in hematopoietic cell differentiation, maintenance and/or function, the single transcript contribution has not been investigated before. Otherwise, very few evidence is currently available on PRDM2. Here, we aimed to analyze the role of *PRDM1* and *PRDM2* different transcripts as mediators of T lymphocyte activation.

**Methods:**

We analyzed the transcription signature of the main variants from *PRDM1* (*BLIMP1a* and *BLIMP1b*) and *PRDM2* (*RIZ1* and *RIZ2*) genes, in human T lymphocytes and Jurkat cells overexpressing *PRDM2* cDNAs following activation through different signals.

**Results:**

T lymphocyte activation induced an early increase of *RIZ2* and *RIZ1* followed by *BLIMP1b* increase and finally by *BLIMP1a* increase. The “first” and the “second” signals shifted the balance towards the PR- forms for both genes. Interestingly, the PI3K signaling pathway modulated the *RIZ1/RIZ2* ratio in favor of *RIZ1* while the balance versus *RIZ2* was promoted by MAPK pathway. Cytokines mediating different Jak/Stat signaling pathways (third signal) early modulated the expression of *PRDM1* and *PRDM2* and the relationship of their different transcripts confirming the early increase of the PR*-* transcripts. Different responses of T cell subpopulations were also observed. Jurkat cells showed that the acute transient *RIZ2* increase promoted the balancing of *PRDM1* forms towards *BLIMP1b*. The stable forced expression of *RIZ1* or *RIZ2* induced a significant variation in the expression of key transcription factors involved in T lymphocyte differentiation. The *BLIMP1a/b* balance shifted in favor of *BLIMP1a* in *RIZ1*-overexpressing cells and of *BLIMP1b* in *RIZ2*-overexpressing cells.

**Conclusions:**

This study provides the first characterization of *PRDM2* in T-lymphocyte activation/differentiation and novel insights on *PRDM1* and *PRDM2* transcription regulation during initial activation phases.

**Supplementary Information:**

The online version contains supplementary material available at 10.1186/s12967-023-04066-x.

## Background

The activation and programming of T cells from their naïve/resting state is a critical point for the functions of the adaptive immune response. This process, characterized by widespread changes in chromatin accessibility triggering extensive changes in transcriptional programs, is orchestrated by a plethora of signals including cytokines, inflammatory and immune-modulatory products, tissue-specific and transcription regulators that ultimately influence the developmental choices of the T cell [1].

PRDF1 and RIZ1 homology domain containing proteins (PRDMs) are a family of structurally related transcriptional regulators, controlling several biological processes, including differentiation of a variety of cell types [2]. Their protein structure exhibits several zinc fingers preceded by the PR/SET domain, which is a SET-like domain typical of methyltransferases [3, 4]. PRDMs function in epigenetic regulation of gene expression through their intrinsic histone methyltransferases (HMTase) and/or via interactions with chromatin modifying enzymes. For instance, no methyltransferase activity has been demonstrated for PRDM1, which achieves its repressor activity through other proteins, including histone deacetylases [5–9] whereas the conserved N-terminal PR domain of PRDM2/RIZ1 has an intrinsic H3K9 methyltransferase activity [10, 11]. *PRDM* genes display a characteristic yin-yang expression pattern where alternative splicing or different promoter use rises to a full-length protein (PR +) and a shorter isoform lacking the PR domain (PR −) [12–14]. An imbalance of these isoforms is often observed in several malignancies, including leukemias and lymphomas, with the PR + product commonly lost or downregulated both by genetic inactivation or epigenetic silencing and the PR − isoform always expressed at high concentration levels [12–14].

**Fig. 1:**
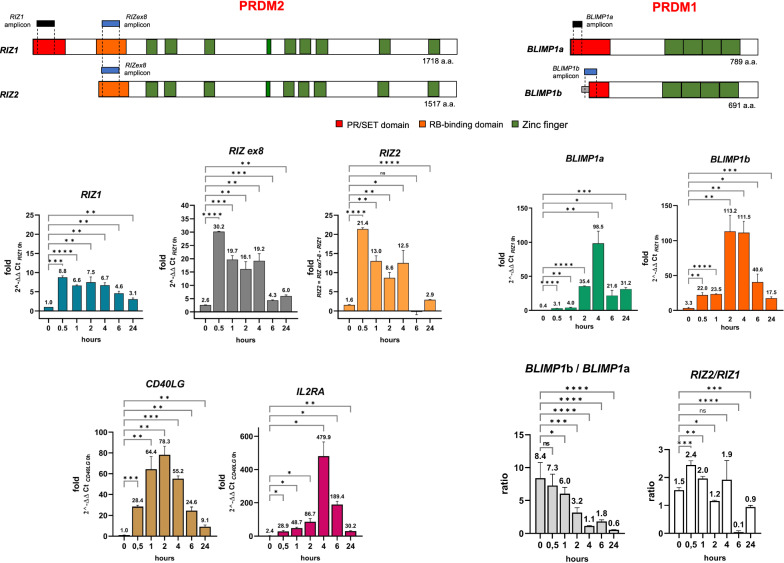
*PRDM1* and *PRDM2* expression in CD4 + T lymphocyte activation. Bar graphs represent data from qRT-PCR analysis of *PRDM1* and *PRDM2* main transcripts (*BLIMP1a, BLIMP1b, RIZ1, RIZ2*) in CD4 + T cells stimulated with anti-CD3/CD28 antibodies. *PRDM1* and *PRDM2* expression levels in CD4^+^T cells were calculated using the ΔΔCt method with the indicated control gene. A schematic illustration of human *PRDM1* and *PRDM2* main products and used amplicons is also reported. To amplify *PRDM1* two sets of primers, recognizing specific sequences of *BLIMP1a* and *BLIMP1b* transcripts, were used. *PRDM2* gene expression was verified using two sets of primers recognizing sequences exclusive of *RIZ1* or a common region to both *RIZ1* and *RIZ2* (and indicated as *RIZex8*). *RIZ2* transcript was measured by subtraction as previously described [46]. The ratio between the PR- and PR + transcripts for each gene was also calculated. The relative expression of activation marker genes *CD40LG* and *IL2RA* was also measured. *p < 0.05, **p < 0.01, ***p < 0.001, ****p < 0.0001

*PRDM1* gene*,* widely known as *BLIMP1* (B lymphocyte-induced maturation protein 1), encodes for two PR + and PR − major isoforms designated as *BLIMP1a* and *BLIMP1b* respectively, which arise from alternative promoter usage. Indeed, *BLIMP1b* is transcribed from a promoter and exon positioned upstream of exon 4 of the *PRDM1* gene. The BLIMP1b protein lacks the first 101 amino acids of BLIMP1a N-terminal region and instead contains three amino acids fused to residues 102–789 of BLIMP1a (see scheme in Fig. 1) [15]. Currently, a lot of studies support its critical role in differentiation, maintenance and/or function of multiple hematopoietic cells of the myeloid and lymphoid lineages [15]. BLIMP1a orchestrates plasma cell differentiation by repressing genetic programs associated with the germinal center stages, while at the same time activating those programs associated with plasma cell functions. Specifically, PRDM1/BLIMP1 drives plasma cell differentiation by repressing several transcription factors, such as Myc, CIITA (MHC class II trans-activator), PAX5 (paired box 5), which is required for B cell fate specification, and BCL6 (B cell lymphoma 6), which promotes germinal centre B cell identity [16–18]. Notably, BCL6 also acts as a transcriptional repressor and forms a negative loop with PRDM1/BLIMP1 [17]. Besides, *PRDM1* expression is induced in activated CD4^+^ and CD8^+^ T lymphocytes where it regulates many aspects of their differentiation in effector and memory cells [19]. Indeed, PRDM1/BLIMP1 also acts as a repressor in subsets of effector CD4^+^ and CD8^+^ T cells [20–26]. As for B cells, a reciprocal repression of BCL6 and PRDM1 is crucial for T lymphocytes differentiation. In this cell context, PRDM1/BLIMP1 represses IL-2 (interleukin 2). IL2 regulates the initial expansion of naïve T cells then, acting through STAT5 (signal transducer and activator of transcription 5), it induces *PRDM1* expression creating a feedback loop that downregulates its own expression during the later stages of T-cell differentiation [27, 28]. PRDM1/BLIMP1 can act also as a transcriptional activator in regulatory T cells by promoting H3K4 methylation at the *IL10* (interleukin 10) locus [20]. A BLIMP1a/BLIMP1b imbalance has been detected in several malignancies derived from B, T, and NK cells [reviewed in 12, 29]. Different regulatory mechanisms, both transcriptional and posttranslational, have been reported to control *PRDM1/BLIMP1* expression. Numerous transcription factors, including BCL-6, PAX5, and BACH2, as well as TNFR and cytokine signaling pathways, are known to directly affect *PRDM1* transcription [30–34].

As *PRDM1*, also *PRDM2* gene expresses two main proteins, PRDM2/RIZ1 (PR +) and PRDM2/RIZ2 (PR −), starting from transcripts initiated by two alternative promoters (see scheme in Fig. 1). *PRDM2* is expressed in immature progenitor cells and in mature myeloid cells [35]. A selective *PRDM2/RIZ1* expression was correlated with myeloid cell differentiation, suggesting a pivotal role for *PRDM2* gene products in the proliferation/differentiation transition [36]. Loss of heterozygosity, promoter hypermethylation or *PRDM2* gene mutations have been observed in several human cancer types, including Diffuse Large B-Cell Lymphoma (DLBCL) [10, 37]. For example, Riz1 KO mice, which retain Riz2 expression, show a high incidence of DLBCLs and other rare non-hematopoietic cancers [37]. Furthermore, *PRDM2* loss is associated with the chronic myelogenous leukemia (CML) collapse to blast crisis [35]. Noteworthy, several reports indicated that PRDM2/RIZ1 is endowed with tumor suppressor activities, whereas PRDM2/RIZ2 acts as an oncogene with putative intrinsic growth-promoting properties [10, 38].

Although previous findings suggest that PRDM2 proteins could have a role in the regulation of the proliferation/differentiation transition in myeloid cells, more extensive investigations in other models (e.g. lymphocytes, hematopoietic stem cells) are advised to confirm a general role of *PRDM2* products in the transition from proliferative activity to a quiescent/differentiate state and vice versa [35, 36].

The aim of this study was to evaluate the mechanisms that regulate the expression of the main molecular variants encoded by *PRDM1* (BLIMP1a and BLIMP1b) and *PRDM2* (RIZ1 and RIZ2) genes, both in lymphocytes from healthy donors and in the Jurkat cell line. Specifically, we aimed to investigate the possible action of RIZ1 and RIZ2 on the gene expression regulation of *BLIMP1a* and *BLIMP1b* transcripts and their role in lymphocytes maturation as effectors of the immune response.

## Methods

### ***Isolation, purification, and stimulation of human CD4***^+^***and CD8***^+^***cells***

Human peripheral T lymphocytes were obtained from total peripheral blood mononuclear cells (PBMCs) collected from 10 human samples (60–200 ml, depending on the experimental needs) of 4 healthy donors (authors who contributed to the present manuscript). PBMCs were isolated by density gradient centrifugation (400*xg* for 30’ at room temperature) on FICOLL-Paque PLUS (GE healthcare Amersham) in 50 mL tubes. After centrifugation the layer of PBMCs was carefully removed and transferred to a new conical tube. Cells were diluted with RPMI-1640 and centrifuged at 200*xg* for 5’ to remove platelets.

To obtain T naïve cells, PBMCs were resuspended in RPMI-1640 and then transferred to PERCOLL (GE healthcare) 40% and 35%. After centrifugation at 700*xg* for 25 min at room temperature, cells were washed, suspended in an appropriate volume of RPMI-1640 and counted. T naïve cell concentration was about 6–7 × 10^6^/mL.

To obtain CD4^+^ and CD8^+^ T lymphocytes, T naïve cells were incubated for 20’ with specific RosetteSep™ (STEMCELL Technologies), diluted with RPMI-1640 (1:2) and then transferred to FICOLL-Paque PLUS (GE healthcare Amersham) in 50 mL tubes. Isolated cells were analyzed by 6 Color TBNK + Truc assay with BD FACSLyric™ flow cytometer (Becton Dickinson) (Additional file 1: Fig S1).

Isolated cells were maintained in RPMI-1640 medium supplemented with 5% fetal bovine serum (FBS) at 37 °C with 5% CO_2_ in a humidified atmosphere. For kinetic analysis, purified CD4^+^ T cells from PBMC were grown in RPMI-1640 25% homolog serum.

### T lymphocyte activation and treatments

To induce T lymphocyte activation, 1 × 10^6^ cells were grown in RPMI-1640 5% FBS at 37 °C with 5% CO_2_ in a humidified atmosphere supplemented with polyclonal activators 50 ng/mL phorbol 12-myristate 13-acetate (PMA) plus 1 μM Ionomycin A (Ion A) (Sigma Aldrich) for 2 h and 6 h. Activation of T cells receptor (TCR) was carried out with antibodies anti-CD3 and anti-CD28 bound to magnetic beads (Dynabeads™, Thermofisher). The percentage of activated cells was measured at 48 h by BD FACSLyric™ flow cytometer (Becton Dickinson) with BD Multitest™ CD8/CD38/CD3/HLA-DR and anti-CD4 PECy7 and anti-CD45 V500C (Becton Dickinson) (Additional file 1: Fig S1).

The mean percentage of activated cells was 33% (SD =  ± 8,2%).

RPMI-1640 was supplemented with IL-2 (30U/mL)(Merck), IL-4 (200 ng/mL), IL-6 (200 ng/mL), IFN-α (180 ng/mL) and IFN-γ (200 ng/mL)(GIBCO™, Thermofisher) where described.

PI3K inhibitor, LY294002 (Ly) or Wortmannin (Calbiochem), were used at final concentration of 10 μM and 1 µM, respectively. The MEK1/2 inhibitor PD98059 (PD) and the Src kinase inhibitor PP1 (Alexis) were used at a final concentration of 5 µM.

### Jurkat cell culture and transfection

Jurkat E6.1 (CLS Cell Lines Service GmbH) cells were grown at 37 °C with 5% CO_2_ in humidified atmosphere in RPMI-1640 supplemented with 10% FBS, 2 mM L-glutamine, and antibiotics (100 U/mL penicillin, 100 mg/mL streptomycin).

The plasmid expressing RIZ1 and RIZ2, were indicated as pSG5_hRIZ1 and pSG5_hRIZ2, respectively. pSG5_hRIZ1 was obtained by cloning in BamH1 site the RIZ1 cDNA from pGEX-RIZ [39] in the FLAG-pSG5 vector. The RIZ2 insert was obtained by PCR amplification of the RIZ1 cDNA sequence with the enzyme PfuTurbo DNA Polymerase (Stragene) using a forward primer containing the BamH1 site and the RIZ2 codon start site and a reverse primer, RIZ2-R, with the stop coding sequence and the BamH1 site (see primer table). To select stable clones of Jurkat cells transfected with pSG5_hRIZ1 and pSG5_hRIZ2, the plasmid pEGFP-C1 (CLONTECH-TaKaRa) was added to the transfection mixture (1:30 compared to FLAG-pSG5, pSG5_hRIZ1 and pSG5_hRIZ2). The EGFP expression was used to evaluate the transfection efficiency and for the resistance to the antibiotic Geniticin G418 (Sigma-Aldrich).

Plasmids for transfection were prepared with Plasmid Midi Kit (Qiagen), according to manufacturer’s instructions.

Cells were transfected using Lipofectamine™ 2000 Reagent in OptiMem I Reduced Serum Medium (Invitrogen™, Thermofisher) for 6 h, following the manufacturer’s instructions. Reaction mixtures were removed, and cells were cultured for an additional 24 h in RPMI1640 with 15% FBS. For Jurkat E6.1 stable clones, selection was performed by addition of 0.8 mg/ml Geneticin G418 (Sigma-Aldrich). pSG5_hRIZ2 shows an epitope tagged by replacing the terminal stop codon with nucleotides encoding FLAG residues (MDYKDDDDK) by PCR. FLAG-pSG5 vector was used as a control together with pEGFP-C1, used as a transfection marker.

### RNA extraction, Semi-Quantitative and Quantitative Reverse Transcriptase Polymerase Chain Reaction (RT-PCR)

Total RNA was extracted from cells using Trizol solution (Invitrogen™, Thermofisher), according to the manufacturer's instructions. The quality and quantity of RNA was assessed by denaturing agarose gel electrophoresis and by spectrophotometry analysis (NanoDrop Technologies), after RNAse-free DNAse-I treatments (Invitrogen™, Thermofisher). RNA was reverse transcribed with SuperScript III (Invitrogen™, Thermofisher) using 500 ng of total RNA; 1 μl of cDNA (60 ng) was used as a template in a PCR reaction with 2.5 U AmpliTaq-Gold (Applied Biosystem™ Termofisher) as previously described [40]. The amplification products were also analyzed by agarose gel electrophoresis. Glyceraldehydes-3-phophate dehydrogenase (*GAPDH*) was used as housekeeping control gene. Densitometry analysis was performed by ImageJ software and differences were assessed by Student’s t-test with a P < 0.05 considered as significant [40].

Quantitative RT-PCR analysis was performed using the SYBR Green PCR Master Mix (Applied Biosystem™ Termofisher),160 nM of each primer and about 50 ng of cDNA (RNA equivalent) as template in an iCycler thermocycler (Bio-Rad Laboratories). PCR condition were 95 °C for 2 min followed by 45 cycles of 15 s at 95 °C, 60 s at annealing temperature of each primer set and 30 s at 72 °C. All reactions were carried out at least in triplicate for every cDNA template and the melting curves were analyzed to verify the specificity of reaction. The relative gene expression was calculated using the 2^−ΔΔCt^ Pfaffl method [41]. *GAPDH* or *RPS18* were used as housekeeping genes for normalization as indicated [40].

Primer sets were designed with Primer3 (Table S1). BLAST and BLAT algorithms at NCBI (http://www.ncbi.nlm.nih.gov) and at Genome Browser (Santa Cruz) (http://genome.ucsc.edu/cgi-bin/hgGateway) were used for sequence comparison and analysis of transcripts.

### Western Blot assay and Densitometric Analysis

Total cell extracts were separated onto SDS–polyacrylamide gels and blotted onto polyvinylidene difluoride membranes [42]. Western blot analysis was performed as described elsewhere [42] with mouse monoclonal antibody FLAG™ clone M2 (Sigma-Aldrich) and rabbit polyclonal antibody RIZ1 (Abcam Ltd.).

### Statistical analysis

Results are reported as mean ± SD. Three independent experiments in triplicates were performed. GraphPad Prism 9,5 software was utilized to perform Brown-Forsythe and Welch ANOVA tests. Significances were indicated as: *p < 0.05 and particularly significant at **p < 0.01, ***p < 0.001, ****p < 0.0001. Expression correlation analysis was evaluated on the GEPIA2 database (http://gepia.cancer-pku.cn/) using the Spearman correlation coefficient R.

## Results

### Polyclonal activation of T Lymphocytes increases PRDM1 and PRDM2 expression levels

To investigate the role of the main molecular variants encoded by *PRDM1* (BLIMP1a and BLIMP1b) and *PRDM2* (RIZ1 and RIZ2) (see scheme in Fig. 1) in the lymphocyte activation we firstly used human peripheral blood lymphocytes and activated them with T cell polyclonal activator phorbol 12-myristate 13-acetate and ionomycin (PMA/Ion) or with anti-CD3/CD28 antibodies at 2 h and 6 h.

Activation of T lymphocytes involves the expression changes of several molecules, both on their surface and within the cell, which clearly characterize them from naïve T cells and can be evaluated by several methods, including gene transcription analysis [43–45].

In our pilot experiment of RT-PCR analysis, we observed changes of *PRDM1* and *PRDM2* transcription together with the expression modifications of genes encoding for transcription factors and cytokines involved in lymphocyte activation and differentiation (*CD40LG*, *KLF2*, *IL2RA*, *IL2* and *MYC*) (Additional file 1: Fig. S2).

*PRDM2* gene expression was verified using two sets of primers recognizing sequences exclusive of *RIZ1* or a common region to both *RIZ1* and *RIZ2* (and indicated as RIZex8). Because of the extensive similarity between the two gene products, *RIZ2* transcript was measured by subtraction as previously described [46]. Similarly, for *PRDM1* two sets of primers, recognizing specific sequences of *BLIMP1a* and *BLIMP1b* transcripts, were used (see scheme in Fig. 1).

To further detail the early events and the kinetics of *PRDM1* and *PRDM2* main transcripts we evaluated their expression by qRT-PCR analysis in human CD4^+^T lymphocytes (greater than 92,5% of total T cells) activated with anti-CD3/CD28 antibodies at different time points (0, 30’, 1 h, 2 h, 4 h, 6 h and 24 h) (Fig. 1). The expression of *CD40LG* and *IL2RA* was also analyzed to monitor lymphocyte activation (Fig. 1).

An expression level increase in *RIZ1* and *RIZ2* transcripts (at major extend) was observed already at 30’ upon lymphocytes activation. At subsequent time points, the *RIZ1* expression is constant over the time, while *RIZ2* fluctuates until 6 h where no *RIZ2* expression was detected (Fig. 1). *BLIMP1a* is not expressed in basal conditions and its expression grows progressively to reach the maximum expression level at 4 h. After this time point, the *BLIMP1a* slows down and maintains a high expression level than the starting point (Fig. 1). An increase in *BLIMP1b* expression level was detected already 30’ after T cell activation, and gained the maximum expression level at 2 h, which is sustained at 4 h. Thereafter, as *BLIMP1a*, also *BLIMP1b* slows down and maintains a higher expression level than the starting point (Fig. 1). An increase in the expression levels of both *PRDM1* and *PRDM2* has been observed upon 24 h activation (Fig. 1). The *CD40LG* increases rapidly upon activation reaching its maximum expression level at 2 h after the CD3/CD28 stimulation. Subsequently, its expression level progressively decreases maintaining constant level greater than time 0 (Fig. 1). The *IL2RA* expression slowly increases during T cell activation, and it peaks upon 4 h CD3/CD28 treatment. At the following time points *IL2RA* expression remains unchanged and higher than control cells (Fig. 1). In addition, we assessed the ratio among *BLIMP1b*/*BLIMP1a* and *RIZ2/RIZ1*. In basal condition CD4^+^T lymphocytes express prevalently *BLIMP1b*; during CD4^+^T lymphocyte activation *BLIMP1b*/*BLIMP1a* decreases progressively in favor of *BLIMP1a*. In basal conditions, CD4^+^T lymphocytes express predominantly *RIZ2*; during CD4^+^T lymphocyte activation *RIZ2/RIZ1*increases until 1 h where it begins to slow down reaching the lower level at 6 h.

*PRDM2* encodes for several transcripts, which can be distinguished also for the presence of different 3’-ends produced by alternative splicing, although they still need to be experimentally validated (see scheme in Additional file 1: Fig S3) [10]. Thus, we also analyzed these slightly different C- terminal tails during T lymphocyte activation utilizing specific oligonucleotides. Interestingly, the amplicon representing the long tail (*RIZ ex10*) displays the same lower trend as *RIZ1* transcript expression levels during T cell activation whereas the higher expression of short tail amplicon (*RIZ ex9a*) prevalently correlates with *RIZ2* (Additional file 1: Fig S3).

Overall, these data suggested the participation of *PRDM2* transcripts other than *PRDM1* ones in the early stages of lymphocyte activation.

### PI3K and Src modulate PRDM1 and PRDM2 gene expression during T lymphocytes activation

Anti-CD3/CD28 or PMA/Ion treatment prompt the activation of various downstream signaling pathways [47–49]. To figure out the cell signaling pathways activated at cell membrane, transduced within the cell, and targeting *PRDM1* and *PRDM2* gene expression, we employed Ly294002 (Ly) or Wortmannin PI3K inhibitors and the PP1 Src kinase inhibitor to treat T lymphocytes activated with anti-CD3/CD28 antibodies for 2 h and 6 h.

**Fig. 2:**
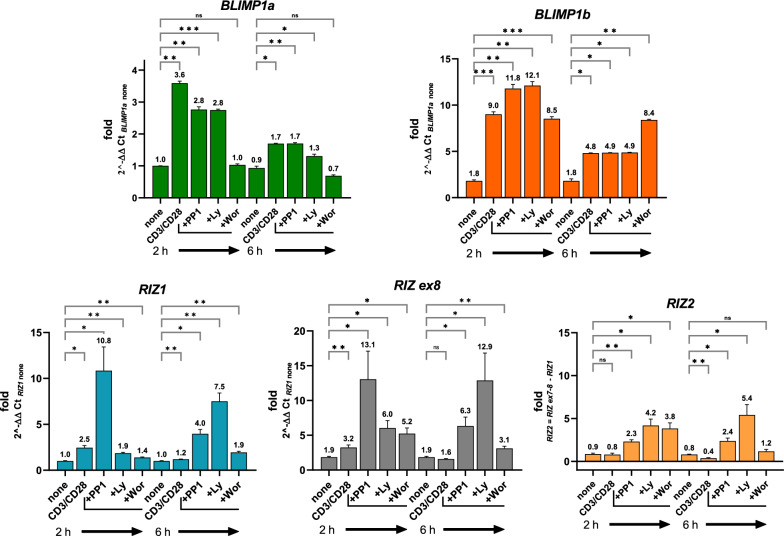
*PRDM1* and *PRDM2* relative gene expression by qRT-PCR analysis in activated T cell treated with Ly, PP1 Src kinase inhibitor and Wortamannin PI3K inhibitor for 2 h and 6 h. *PRDM1* and *PRDM2* transcript levels were compared to their expression in naïve T cells (with expression value equal to 1). *RPS18* was used as control gene. Three independent experiments in triplicates were performed and data expressed as mean ± SD. *ns* (not significant), *p < 0.05, **p < 0.01, ***p < 0.001

The qRT-PCR analysis showed that Ly and Wortmannin treatment reduced the RIZ1 expression levels and increased the total RIZ products expression levels, probably due to a RIZ2 increase, thus reverting the effect induced by T cell activation on RIZ1 and potentiating the effect on total RIZ (*RIZex8*) at 2 h. In addition, Ly and Wortmannin increased both *RIZ1* and *RIZex8* at 6 h. Otherwise, PP1 treatment augmented both *RIZ1* and *RIZex8* expression levels observed upon T cell activation already at 2 h (Fig. 2).

Wortmannin hindered the *BLIMP1a* increase induced at 2 h and 6 h without affecting the *BLIMP1b* expression at 2 h, whereas increased *BLIMP1b* expression levels at 6 h (Fig. 2). Ly prevented the expression level increase of *BLIMP1a* at 2 h and 6 h, potentiated the increase of *BLIMP1b* at 2 h and has not produced any effect on *BLIMP1b* at 6 h. PP1 prevented and potentiated the *BLIMP1a* and *BLIMP1b* increase respectively at 2 h, and has not modified *BLIMP1a* and *BLIMP1b* expression at 6 h (Fig. 2). Altogether, these results suggested that the signaling pathways involving PI3K and Src kinase modulates *PRDM1* and *PRDM2* gene expression during T lymphocyte activation.

### IL2, IL4 and IFNA modify the PRDM1 and PRDM2 gene expression levels in activated T lymphocytes

**Fig. 3:**
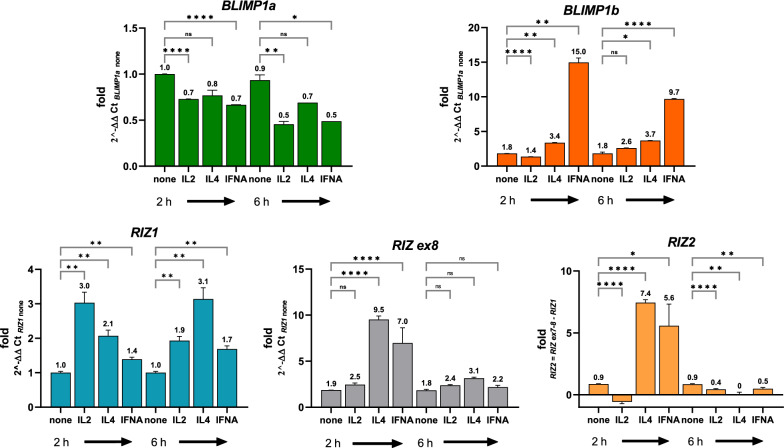
Cytokine treatments regulate *PRDM1* and *PRDM2* gene expression levels in activated T cells. *BLIMP1a, BLIMP1b, RIZ1, RIZex8* and *RIZ2* gene expression analyses by qRT-PCR in activated T cells were compared to naïve T cells after treatment with IL2, IL4 and IFNA at 2 h and at 6 h. Three independent experiments in triplicates were performed and data expressed as mean ± SD. *P < 0.05 *vs* control cells. *p < 0.05, **p < 0.01, ***p < 0.001, ****p < 0.0001

Cytokines trigger intracellular signaling cascades regulating gene expression in target cells, which lead to their activation, proliferation, and differentiation [1, 43]. IL2 provides an additional signal to the antigen recognition that triggers cyclin-dependent kinases and promotes the S phase cell cycle entry through STAT1/STAT3/STAT5 activation [43]. Therefore, IL2 promotes T cell proliferation and differentiation. IL4, by inducing the cell signaling transduction through STAT5/STAT6 and IRS-2 promotes naïve T lymphocyte differentiation in Th2 cells. In addition, IL4 represents the typical growth factor for Th2 cells. IFN-α modulates STAT1/STAT2 activation and prompts Th1 polarization. Thus, we aimed to assess the effect of IL2, IL4 and IFNA on *PRDM1* and *PRDM2* transcriptional levels in T lymphocytes isolated from healthy donor PBMCs. As shown in Fig. 3, IL2 modified *PRDM2* gene expression at 2 h and 6 h, by increasing *RIZ1* expression levels without significantly altering *PRDM2/RIZex8* amount; however, IL2 induced a RIZ2 downregulation at 2 and 6 h. IL4 increased both *RIZ1* and *PRDM2/RIZex8* expression levels at 2 h and 6 h; IFNA upregulated RIZ1at 2 and 6 h and particularly influenced *PRDM2/RIZex8* expression at 2 h revealing a *RIZ2* up-regulation (Fig. 3). As regards to PRDM1, IL2 downregulated both transcripts at 2 h and *BLIMP1a* also at 6 h. IL4 modulated only *BLIMP1b* by increasing its expression at 2 h and 6 h. IFN-α down-regulated *BLIMP1a* and up-regulated *BLIMP1b* at 2 and 6 h (Fig. 3).

### Analysis of PRDM1 and PRDM2 transcripts in different T cell subpopulations

**Fig. 4:**
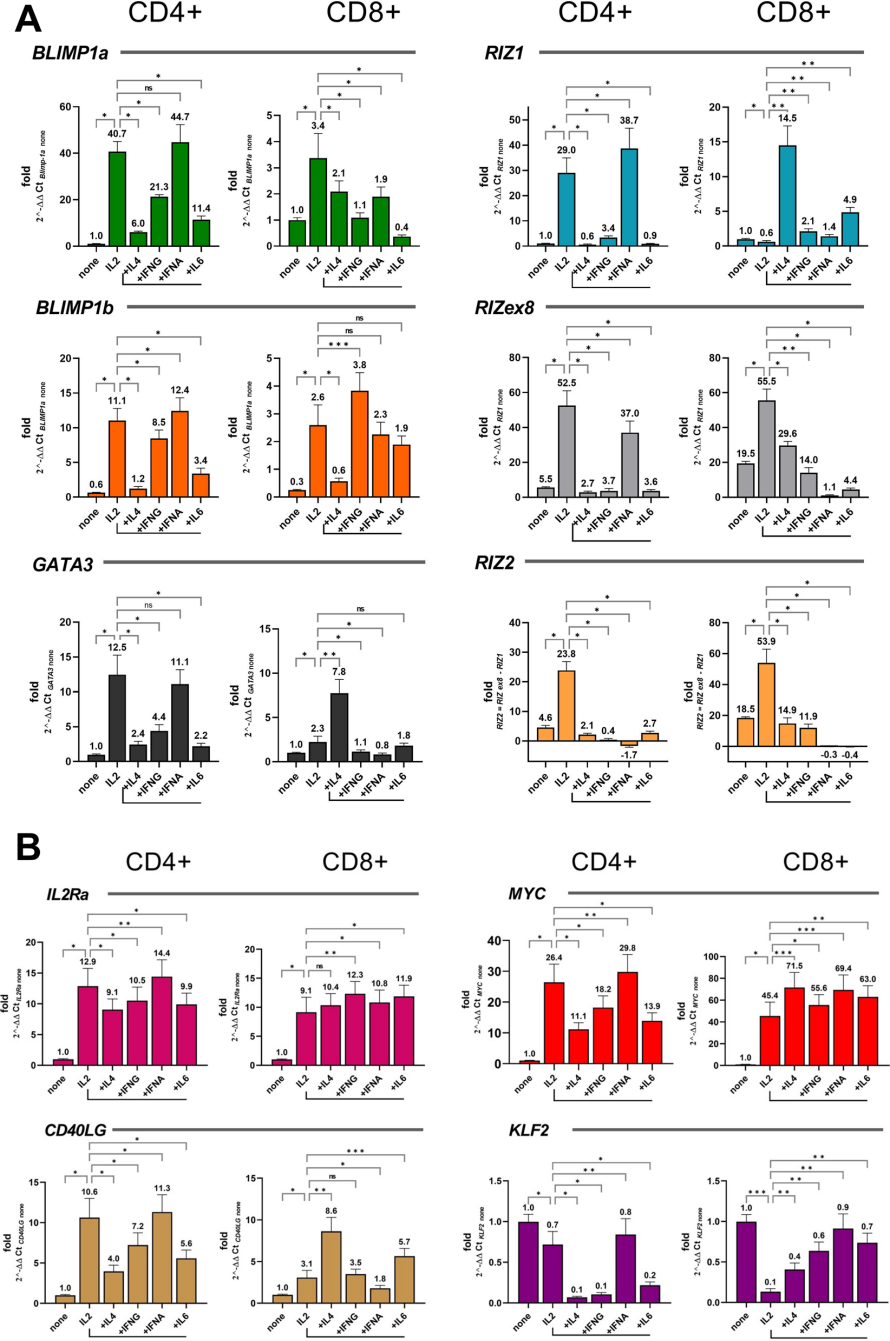
Analysis of different T cell subpopulations. (**A**) *PRDM2/RIZ*, *PRDM1/BLIMP1*, *GATA3* and (**B**) *CD40LG*, *IL2RA*, *MYC* and *KLF2* genes were assayed in CD4^+^ and CD8^+^ positive cells activated with anti-CD3/CD28 antibodies and treated with IL-2 supplemented with IL4, IFNG, IFNA and IL6 for 48 h. As in the previous experiments, PR + and PR- transcripts of *RIZ* and *BLIMP1* were tested (see also Fig. 1 for transcript details). Three independent experiments in triplicates were performed and data expressed as mean ± SD. *ns* (not significant), *p < 0.05, **p < 0.01, ***p < 0.001, ****p < 0.0001

To explore the T cell subset in which polarizing cytokines target *PRDM1* and *PRDM2* genes expression during differentiation induction, lymphocytes were previously separated into naïve CD4^+^ and CD8^+^ T positive cells, activated with anti-CD3/CD28 antibodies and with IL2 supplemented with different further cytokines, as IL4, IL6, IFNA and IFNG for 48 h (Fig. 4A). We also measured the levels of *GATA3*, which is critical for the development, differentiation and function of CD4^+^ T cell subsets, as well as CD8^+^ T cells [50].

Anti-CD3/CD28 and IL2 treatment of CD4^+^ T lymphocytes increased *PRDM2* expression levels. However, IL4, IL6 or INFG cytokines, but not IFNA, were able to dramatically revert the effect of IL2 on *PRDM2* expression level increase (Fig. 4A). Similarly, IL2 treatment of CD4^+^ T lymphocytes produced a consistent increase of both PRDM1 molecular forms, particularly of *BLIMP1a*. All the cytokines supplemented to IL2, excluding IFNA, resulted in a reduction of both *BLIMP1a* and *BLIMP1b* expression level (Fig. 4A).

In CD8^+^ T activated lymphocytes, treatment with IL2 reduced *RIZ1* expression levels and increased *RIZ2* expression level. The other cytokines supplemented to IL2 induced a downregulation of *RIZ2* and an upregulation of *RIZ1*. In CD8^+^ activated T lymphocytes, IL2 treatment upregulated both *PRDM1* transcripts. However, no significant effect was found for *BLIMP1b* expression with the addition of a second cytokine, except for IL4 that reduced its expression. Besides, *BLIMP1a* ever decreased in the presence of a second cytokine.

Interestingly, in the same experiment shown in Fig. 4A, our gene expression analysis in CD4^+^, and CD8^+^ T subpopulations during T cell activation revealed an upregulation of *CD40LG*, *IL2RA*, *MYC* and *KLF2*, paralleling previous literature data [51–54] (Fig. 4B).

Collectively, T cell subpopulations revealed a different response to the “third signal” provided by the several cytokines supplemented to the activated T cells treated with IL2 when we observed the *PRDM* gene expression levels. Indeed, while *BLIMP1a* and *BLIMP1b* variants exhibited a substantial similarity in their trend, *PRDM2* products showed a great variation.

### PRDM1 and PRDM2 expression level analysis during Jurkat cell activation

**Fig. 5:**
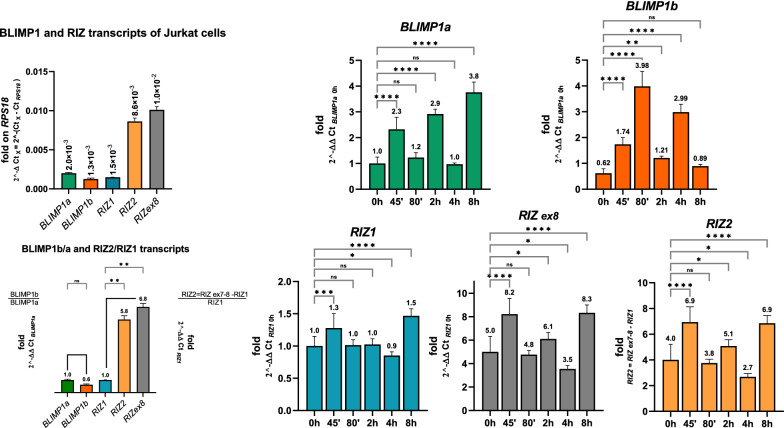
Gene expression analysis of *PRDM*1 and *PRDM2* in Jurkat cells. On the left upper side, the basal levels of *BLIMP1a, BLIMP1b, RIZ1, RIZex8* and *RIZ2* in Jurkat cells are represented as fold on *RPS18* control gene. The other bar graphs represent data from qRT-PCR analysis of the same transcripts in Jurkat cells following activation with PMA/Ion. Expression levels were calculated using the ΔΔCt method with the indicated control gene. Three independent experiments in triplicates were performed and data expressed as mean ± SD. *ns* (not significant), *p < 0.05, **p < 0.01, ***p < 0.001, ****p < 0.0001

The Jurkat cell line is an immortalized T lymphocyte cell line originally obtained from the peripheral blood sample of a young patient with T cell leukemia [55]. The Jurkat cell line has often been used as a prototypical T cell line to study multiple events in T cell biology, including signal transduction pathways involved in lymphocyte activation [56]. Moreover, considering the high difficulties to transfect normal lymphocytes, this cell line is suitable for evaluating the overexpression of genes by transfection. Firstly, we assessed the *PRDM1* and *PRDM2* expression levels in Jurkat cell line by qRT-PCR analysis. In standard culture conditions (see Methods), Jurkat cells expressed prevalently *PRDM2*; besides, they showed similar expression levels of *PRDM1* transcripts whereas *RIZ2* was expressed at higher levels than *RIZ1* (about six folds) (Fig. 5). Then, we assessed the expression levels of *PRDM1* and *PRDM2* transcripts at early time points after activation with PMA/Ion (45’, 80’, 2 h, 4 h and 8 h) in Jurkat cells (Fig. 5). As observed in T cell lymphocytes, both *PRDM1* and *PRDM2* transcripts showed a cyclic expression variation even though with different magnitudes and kinetics. Specifically, in basal conditions and at 45’ the predominant *PRDM2* isoform is *RIZ2* that is downregulated at 2 h and 4 h. At 4 h a decrease of both isoforms was observed. Upon 8 h activation, RIZ1 and RIZ2 expression level is equal to the level observed at 45’ (Fig. 5).

A cyclic variation in *BLIMP1a* and *BLIMP1b* expression level has been observed during lymphocyte activation: both *BLIMP1a* and *BLIMP1b* are upregulated at 45'. *BLIMP1a* increased at 2 h and 8 h while at 80’ and 4 h *BLIMP1a* showed an expression level similar to that observed in basal conditions. An opposite trend has been revealed for *BLIMP1b* at the same time (Fig. 5).

### PRDM2 transcript overexpression in Jurkat cells

Hypothesizing a role of master regulator gene for *PRDM2* in T lymphocyte activation and a hierarchical organization in the expression level regulation of *PRDM* genes, Jurkat cell line was transiently transfected with plasmids encoding for RIZ1 (pSG5_rRIZ1), RIZ2 (pSG5_hRIZ2) and with the empty vector (pSG5) to investigate the ability of RIZ1 and RIZ2 to modulate the gene expression of pivotal factors involved in lymphocyte activation and differentiation. The *RIZ1* and *RIZ2* overexpression was evaluated after transfection by qRT-PCR of reverse-transcribed total cellular RNA and Western blot analysis. qRT-PCR analysis revealed a significant increase of *RIZ1* and *RIZ2* transcripts compared to control cells (pSG5) (Additional file 1: Fig S4). RIZ1 protein level was evaluated by Western blot analysis with the monoclonal antibody recognizing the PR domain exclusive of RIZ1. RIZ2 protein overexpression was revealed using a monoclonal antibody recognizing the Flag epitope. Altogether, both qRT-PCR and Western blot analyses confirmed RIZ1 and RIZ2 overexpression (Additional file 1: Fig S4).

Then, we analyzed if the overexpression of RIZ1 and RIZ2 was able to modulate *PRDM1* expression level and modify the ratio among the *BLIMP1a* and *BLIMP1b* forms.

**Fig. 6:**
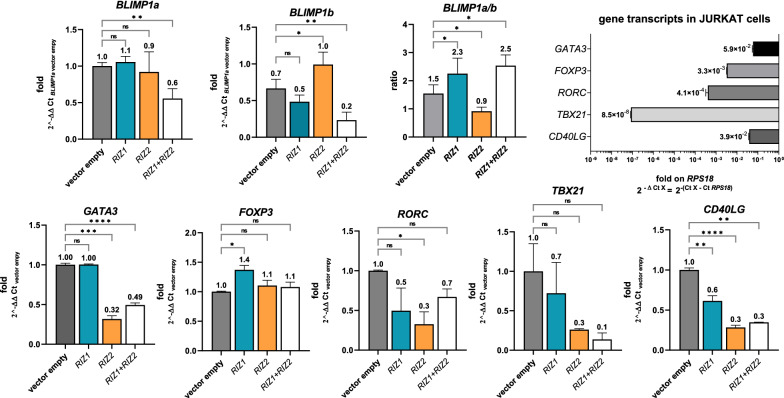
Gene expression analysis of lymphocyte activation related transcription factors in Jurkat transiently *RIZ* overexpressing cells. *PRDM1* transcript levels were compared in Jurkat cells transiently transfected with the indicated encoding plasmids (with the expression value of the empty vector equal to 1). The ratio between the PR- and PR + transcripts for each gene was also calculated. RIZ2 overexpression reduced the ratio between *BLIMP1a* and *BLIMP1b* in favor of *BLIMP1b*. The expression of *GATA3*, *TBX21*, *FOXP3*, *RORC*, *CD40LG* was also measured in Jurkat cells in both basal conditions (ΔCt method as fold on *RPS18* control gene) and after 36 h transient transfection with the plasmid encoding for RIZ1, RIZ2 or with pSG5 control (ΔΔCt method with the indicated control gene). Three independent experiments in triplicates were performed and data expressed as mean ± SD. *ns* (not significant), *p < 0.05, **p < 0.01, ***p < 0.001, ****p < 0.0001.

As depicted in Fig. 6, RIZ1 transient overexpression in Jurkat cell line has not significantly modified *BLIMP1a* and *BLIMP1b* gene expression levels, while RIZ2 overexpression increased *BLIMP1b* without affecting *BLIMP1a* expression levels. When we co-transfected both *RIZ1* and *RIZ2*, we observed a reduction of both *BLIMP1a* and *BLIMP1b* transcripts. Besides, RIZ1 overexpression increased the *BLIMP1a*/*BLIMP1b* ratio also in the presence of *RIZ2* (> 2 folds). RIZ2 alone, instead, reduced the ratio in favor of *BLIMP1b* (Fig. 6).

In standard culture conditions, Jurkat cells exhibited different expression levels of transcription factors involved in lymphocyte differentiation (Fig. 6). Specifically, they expressed high levels of *GATA3*, alike *CD40LG*, moderate levels of *FOXP3* and *RORC* (about 10 folds lower than *CD40LG*) and very low levels of *TBX21* (about 100 folds lower than *CD40LG*). Interestingly, the overexpression of RIZ1 or RIZ2 in Jurkat cells altered the expression of these factors; RIZ2 overexpressing cells showed a significant reduction in *GATA3* expression. In addition, RIZ2 overexpression reduced *RORC* expression level while RIZ1 overexpression has not significantly affected its transcription. In both RIZ1 and RIZ2 overexpressing cells we observed a reduction in *CD40LG* expression, while *FOXP3* was upregulated only in RIZ1 overexpressing cells (Fig. 6). Collectively, RIZ2 forced expression through an imbalance of RIZ1/RIZ2 ratio in favor of RIZ2, induced a *BLIMP1b* increase. Consequently, the balance among *BLIMP1a* and *BLIMP1b* has shifted in disfavor of the first form. Otherwise, a balance in favor of *BLIMP1*a was promoted by RIZ1 overexpression through *BLIMP1*b repression. In conclusion, we speculate that PRDM2 may act upstream PRDM1 by regulating its gene expression. In addition, upon *PRDM2* overexpression the main transcription factors involved in lymphocyte differentiation showed a down-regulation except for FOXP3 that is up-regulated by RIZ1 suggesting a putative PRDM2 involvement in the regulation of lymphocyte differentiation. Meanwhile, RIZ1 and/or RIZ2 could act on *CD40LG* in T lymphocytes activation with a negative feedback mechanism of regulation. *TBX21* was expressed at very low levels; thus, we were not able to appreciate statistically significant variations.

### Polyclonal activation of Jurkat cells stably transfected with RIZ1 and RIZ2

**Fig. 7:**
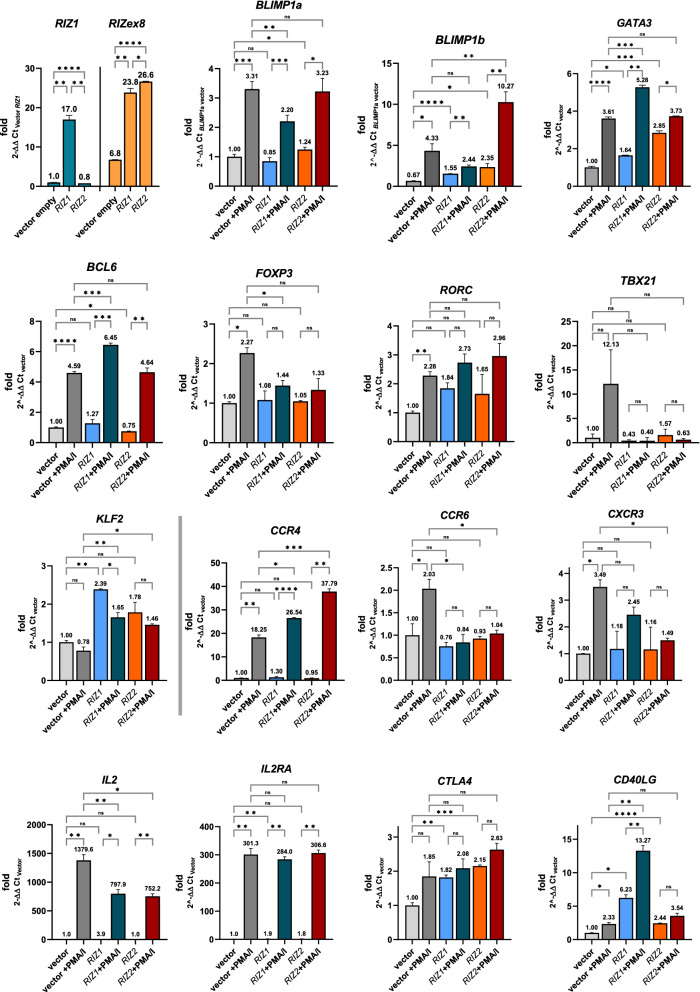
A Gene expression analysis of the main transcription factors related to lymphocyte activation in Jurkat stably transfected cells. qRT-PCR analysis of *BLIMP1a*, *BLIMP1b*, *GATA3*, *FOXP3*, *RORC*, *TBX21*, *BCL6*, *CD40LG*, *KLF2*, *CTLA4*, *IL2*, *IL2RA*, *CCR4*, *CCR6*, and *CXCR3* on Jurkat cells transfected with the plasmid encoding for RIZ1, RIZ2 or with pSG5 control vector and treated with or without treatment with polyclonal activators for 6 h. Three independent experiments in triplicates were performed and data expressed as mean ± SD. *ns* (not significant), *p < 0.05, **p < 0.01, ***p < 0.001, ****p < 0.0001

To investigate the possible effect of stable overexpression of *PRDM2* molecular variants on target genes, qRT-PCR analysis was performed in Jurkat cells stably transfected with the plasmid encoding for RIZ1 (pSG5_rRIZ1), RIZ2 (pSG5_hRIZ2) or with pSG5 control vector (see Additional file 1: Fig S4 for details). In non-activated cells, only RIZ2 stable overexpression increased *BLIMP1a* level, as shown in Fig. 7. Instead, both RIZ1 and RIZ2 increased *BLIMP1*b expression levels. In addition, RIZ2 significantly increased the expression level of the main transcription factor of lymphocyte differentiation, *GATA3*, a similar effect as observed for *BLIMP1*b, while RIZ1 increased it to a lesser extent. Besides, a reduction of *BCL6* expression was detected in RIZ2 overexpressing cells. No significant changes were observed for *FOXP3, RORC* and *TBX21. CD40LG* was noticeably increased by RIZ1, and to a less extent by RIZ2.

Activation of Jurkat cells stably transfected with pSG5_rRIZ1 or pSG5_hRIZ2 increased the level of both *PRDM1* transcripts compared to control cells. RIZ1 overexpression reduced the *BLIMP1*a and *BLIMP1*b expression increase induced by cell activation, particularly for *BLIMP1*b. RIZ2 overexpression did not affect the *BLIMP1*a expression level whereas significantly amplified *BLIMP1*b expression increase observed after activation (of about 2.5 folds) (Fig. 7).

The RIZ1 overexpression prevented the *FOXP3* expression level increase observed upon activation. No significant variations were detected for *TBX21*. Only the RIZ1 overexpression augmented *GATA3* expression level increase observed in Jurkat cell activation. Despite its elevated basal level, *CD40LG* showed a conserved inducibility with a markedly higher increase of total levels in RIZ1 overexpressing cells. By observing the expression of genes encoding chemokine receptors related to the effector functions of different lymphocyte subsets, the *CCR4* gene was significantly increased in activated RIZ1 and RIZ2 overexpressing cells. Instead, *CCR6* was repressed by both RIZ1 and RIZ2 while *CXCR3* was repressed only by RIZ2 (Fig. 7). Moreover, RIZ1 and RIZ2 overexpression did not modify the *IL2* levels in basal conditions but halved the *IL2* upregulation observed in activated lymphocytes. RIZ1 overexpression increased *IL2RA* levels in basal conditions. Both RIZ1 and RIZ2 overexpression did not significantly modify *IL2RA* upon lymphocyte activation. RIZ1 and RIZ2 overexpression increased the basal level of CTLA4 that was not modulated during lymphocyte activation (Fig. 7).

### PRDM1 and PRDM2 transcripts positively correlate with expression of T cell activation genes

**Fig. 8:**
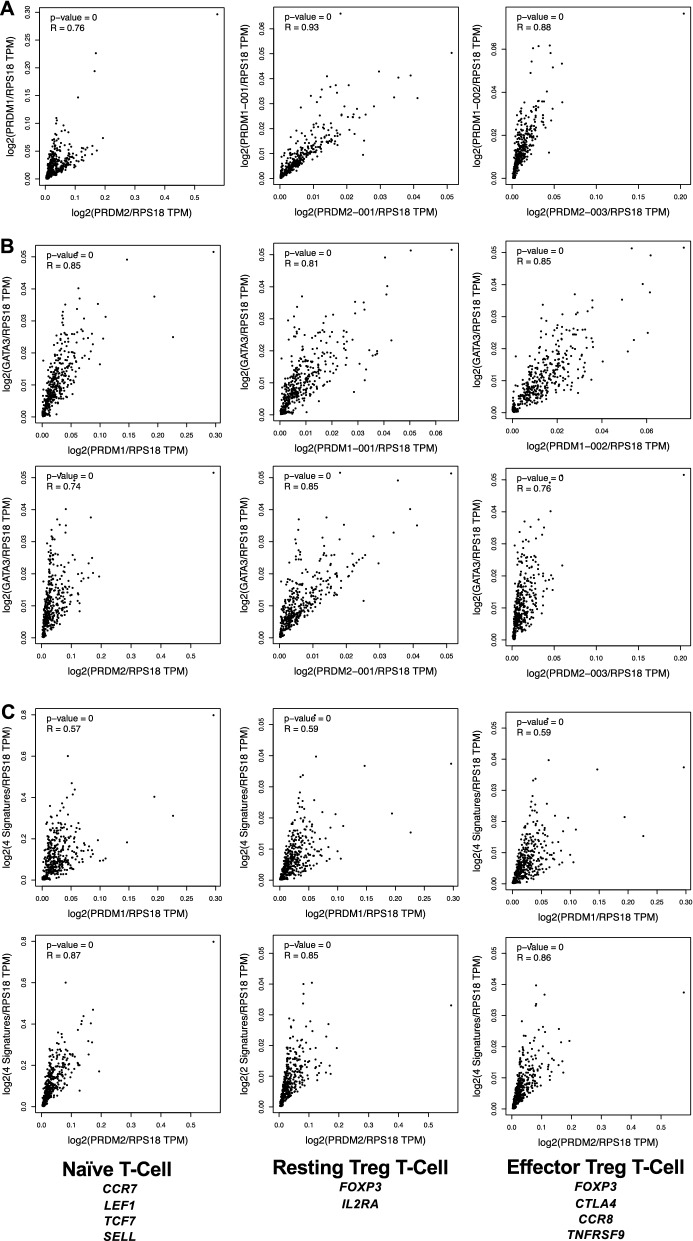
Expression correlation analysis of *PRDM1*, *PRDM2* and the main transcripts with the indicated gene/transcript was performed on GEPIA2 platform using the Spearman correlation coefficient R. Datasets evaluated: whole blood, thymoma normal and EBV-transformed lymphocytes. PRDM2-001 from GEPIA2 database represents *RIZ1* transcript, PRDM2-003 represents *RIZ2* transcript, PRDM1-001 represents *BLIMP1a* transcript and PRDM1-002 represents *BLIMP1b* transcript. Scatter plots of mRNA levels were normalized by *RPS18* expression. **A** Correlation analysis between *PRDM1* and *PRDM2* gene expression; **B** Correlation between PRDM1, PRDM2 and their main transcripts with *GATA3*; **C** Correlation between *PRDM1*, *PRDM2* and their main transcripts with the indicated signatures

To further support the relationship between *PRDM1* and *PRDM2* and lymphocyte activation we explored the expression of their transcripts by in silico analysis of GEPIA2 database [57]. Specifically, we measured the Spearman correlation coefficient R to evaluate the expression correlation of *PRDM1*, *PRDM2* and their main transcripts on three datasets from The Cancer Genome Atlas (TCGA) and Genotype-Tissue Expression (GTEx): whole blood, thymoma normal and EBV-transformed lymphocytes. The mRNA levels of the analyzed genes were normalized by *RPS18* expression. As depicted in Fig. 8A, a high positive correlation was found between *PRDM1* and *PRDM2* (R = 0.76) when their whole transcription was considered. When we analyzed the PR + (*RIZ1* and *BLIMP1a*) and PR- (*RIZ2* and *BLIMP1b*) transcripts, we found a stronger correlation in both cases (R = 0.93 and R = 0.88 respectively). Interestingly, high correlations were also observed between *PRDM1* and *PRDM2* transcripts with *GATA3* (R > 0.7) (Fig. 8B). Again, when we matched the whole *PRDM1* or *PRDM2* transcripts with gene signatures of immune response, a moderate correlation was found for *PRDM1* (R ≥ 0.57) whereas a high correlation resulted for *PRDM2* (R ≥ 0.85) (Fig. 8C).

## Discussion

PRDM family genes play a pivotal role in the control of several aspects of cell behavior, such as cell cycle progression, cell development and differentiation as well as the homeostasis maintenance of immune system cells [2, 3, 13]. The lymphocyte differentiation is mainly due to histone modifications caused by methyltransferase proteins [58, 59]. A very close relationship between *PRDM* gene expression and lymphocyte function regulation could exist [60]. To date, several studies have discussed the PRDM1 pivotal role in lymphocyte activation and differentiation. Indeed, BLIMP1 protein is considered an essential regulator of terminal B cell differentiation into antibody-secreting plasma cells and controls the differentiation of Th1/Th2 cells [15, 61]. PRDM2 products are expressed in T lymphocytes where they interact with GATA-3, an essential transcription factor for T cell development and the Th2 lymphocyte differentiation, thereby suggesting that PRDM2 regulates GATA-3 function [62]. The role of PRDM2/RIZ in lymphocytes has not been elucidated so far. Several findings highlighted the relationship between PRDM2 and proliferation control, represented by the Yin-Yang paradigmatic model. Here, we have investigated the role of *PRDM2* gene and its molecular variants using the human peripheral blood lymphocytes. Indeed, normal lymphocytes represent a good model to evaluate events induced by mitogenic signals, understand their control mechanisms than tumor cell lines or cells transformed with oncogenes. We have analyzed the transcription of *PRDM1* and *PRDM2* variants through qRT-PCR because of the high specificity and sensitivity of this method. Besides, it represents a proper tool to study the early events of T cell activation. Indeed, it is well established that distinct transcriptional profiles and chromatin modifications are observed during T cell response [63].

Our findings represent the first evidence on the putative role of PRDM2 proteins (RIZ1 and RIZ2) in the commitment of T lymphocytes to the functions subsequent to their activation. Moreover, as regard to PRDM1, we have also characterized the expression of the different *PRDM1/BLIMP1* (a and b) transcripts during activation. Specifically, we have analyzed the transcription signature of these genes in both human peripheral blood T lymphocytes and Jurkat cells after activation operating through the “first” signal mediated by TCR, and the “second” signal triggered by CD28, or through the cell signaling transduction pathways downstream the receptors. Noteworthy, our findings are also supported by in silico data on GEPIA2; indeed, gene expression correlation analyses clearly indicated a high correlation between *PRDM1* and *PRDM2*, and between their transcripts and other recognized genes involved in immune response (Fig. 8).

We observed that the activation of PBMC or naïve CD4^+^ T lymphocytes induced an increase in the expression levels of the *PRDM2/RIZ* and *PRDM1/BLIMP1* genes, with a correlated increase of *MYC* gene expression, a target of both PRDM1 and PRDM2 proteins [64, 65] (Fig. 1). The expression profile of *PRDM1* and *PRDM2* variants over the time showed the early increase of *RIZ2* and *RIZ1* followed by *BLIMP1b* increase and finally by *BLIMP1a* increase. The “first” and the “second” signals shifted the balance between the PR + and the PR- forms in favor of the PR- ones, for both studied genes. Furthermore, we have also evidenced that the short tail amplicon (*RIZ ex9a*) prevalently correlates with *RIZ2* transcription (Additional file 1: Fig S3); however, further studies are still needed to fully elucidate the functional role of these *PRDM2* transcription variants [10].

These genes showed a trend similar to that of the Immediate Early Growth Response Genes, which determine important changes in the cellular state of Jurkat T cells [66]. Specifically, they regulate the switch of resting lymphocytes from quiescent to activated state, followed by cell proliferation essential for clonal expansion and subsequent differentiation. Furthermore, to determine the role of the first and second signal transduction pathways, TCR and CD3/CD28 respectively, in the gene expression regulation of *PRDMs*, the effects of several inhibitors of key enzymes of transduction pathways were evaluated, such as PI3 and MEK1/2 kinases. The obtained results suggested that the signaling pathway involving PI3K modulates the RIZ1/RIZ2 ratio in favor of *RIZ1* gene during T lymphocyte activation while the balance versus *RIZ2* was promoted by the MAPK pathway. Similarly, in the CML model, RIZ1 was induced from myeloid cells, where PI3K/AKT upregulates *RIZ1* [67].

The presence of cytokines that mediate different Jak/Stat signaling pathways are observed as agents capable of modulating the expression of *PRDM1/PRDM2* genes and the relationship of their forms (Fig. 2). IL-2/STAT5 promotes the increase in the *RIZ1* amount compared to *RIZ2*; instead, IL-4/STAT6 induces an increase of *PRDM2/RIZ*, most likely of the *RIZ2* form. Besides, IFN-∝/STAT1/2/IRF9 produces the most marked increase in *RIZ2*. All these effects seem to be very quick, already at 2 hrs whereas at 6 hrs we observed non-significant variation in expression. In the same T cells, stimulated as above, we observed an early increase in the *BLIMP1b* form with IFN-∝ and late as with IL-2 or IL-4, while the increase in *BLIMP1a* is only late. The relationship between the *PRDM1* forms towards the form *a* is evident in the presence of IL-2 and IL-4 while with IFN-∝ the increase in the form *a* balances the form *b*. Overall, this analysis would confirm what we observed with the activation of the 1st and 2nd signals, i.e. the early increase of the PR*-*minus transcripts compared to the PR-plus ones. The physiological activation by the antigen presenting cell (APC) generates clonal expansion by means of 1st and 2nd signals and the 3rd signal, which is prompted by the presence of “inducing” cytokines produced by different innate cells present in the microenvironment or by the APCs themselves, promotes the differentiation of T cell subtypes. The presence of all the three signals, simulated in the CD4^+^ T cells and CD8^+^ T cells populations, involves a highly different expression trend between *PRDM2* and *PRDM1* for many of the cytokines added, such as IL-4, IFN-γ, IFN-∝ and IL-6, to the main stimulus CD3/CD28 + IL-2 (Fig. 4). In summary, it is likely that in CD4^+^ cells IL-4 produces a reduction in the induction of *PRDM1* and a repression of *PRDM2*. The latter effect is also observed with other pro-differentiating cytokines, such as IFN-γ and IL-6. In CD8^+^ T cells, the *RIZ2* and *BLIMP1b* increase occurs through the activation with IL-2 and CD3/CD28 alone, while the presence of cytokines pushes the prevalence of *RIZ1* (IL-4) or the repression of *RIZ2* (IFN-∝ and IL-6). On the other hand, *BLIMP1b* increases, and in the presence of IL-4, a balance is produced towards *a* while *b* form is repressed. In the presence of the other cytokines, IFN-γ, IFN-∝ and IL-6, the negative regulation of *a* form promotes a greater balance towards the *b* form. From these findings, we deduce that the response of the CD4^+^ or CD8^+^ effector T cells is different regarding the expression of the *PRDM1* and *PRDM2* genes and their variants, if we compare the trend of T cell activation markers (*KLF2*, *CD40LG*, *IL2RA*, *MYC*). The used model of enriched polyclonal human T cells, which represent the sum of different T subsets (Th1, Th2, TH17, TFh etc.), does not allow a precise assessment of the associations between *PRDM1* and *PRDM2* transcripts and the different cytokine stimuli, but these data solicit further correlation and functional studies to better understand the cause/effect role of *PRDM1* and *PRDM2* and their variants in the mechanism of action of the cytokines themselves.

These observations highlight the relationship between stimulus and response downstream of the *PRDM1* and *PRDM2* genes and of the different expressivity of their variants. Thus, the question is how and in what way the different pathways are concatenated and if there could be a cause-effect hierarchy between the early activation of *RIZ1* and *RIZ2* and the late activation of *BLIMP1a* and *BLIMP1b*. To this purpose, we performed overexpression experiments of *PRDM2/RIZ* isoforms in Jurkat cell line (although they already overexpress *RIZ2* at baseline condition) to reproduce significant variations of RIZ1 and RIZ2 as those induced by the 1st/2nd signals over time and observed in T cells from PBMCs and assess the effects on *BLIMP1a* and *BLIMP1b* expression levels. This approach allowed us observing that the acute (transiently) increase of *RIZ2* promotes the balancing of the *PRDM1* forms towards the *b* form (Fig. 5). Noteworthy, the generation of stable Jurkat clones overexpressing *RIZ1* or *RIZ2* allowed us to evidence a different equilibrium of the *BLIMP1a/b* molecules in basal conditions beyond the modification of the response to the polyclonal activators (Fig. 6). The RIZ2-mediated adaptation of the cells led to a basal increase in *BLIMP1b* and preserved its induction by the 1st/2nd signals. Obviously, through an autocrine mechanism, cells can promote the expression of *IL2,* which is not expressed in basal conditions in the different stable RIZ1 or RIZ2 overexpressing cell lines. Stable RIZ1 cell line promotes a lower increase in the baseline *BLIMP1b* level and, on the other hand, a strong repression effect of the 1st/2nd signal induction, thus highlighting the importance of RIZ1 and RIZ2. *GATA3* gene appears strongly modulated under basal conditions in RIZ2 overexpressing cells in a similar manner as *BLIMP1b*. Otherwise, in RIZ1 overexpressing cells, *GATA3* increases little in basal conditions but responds to activation stimuli. This evidence confirms the hypothesis of a close relationship between PRDM2 and GATA3, and it indicates significant differences between the action of the *PRDM2* forms on the *GATA3* expression regulation. Different changes of other analyzed genes (*FOXP3, RORC, BCL6*, etc.), compared to *BLIMP1* and *GATA3*, seem to confirm the different activity of the PRDM2 forms, RIZ1 and RIZ2, both in the increase or decrease of the basal amount and in the induction capacity by the activation signals. This finding is in line with the basal *GATA3* increase, which predisposes to the cell function increase in the T helper 2 direction. Likely, RIZ2 could work in concert with GATA3 to strengthen its functions, including the modulation of *CCR4* expression. Instead, comparing the effects on other key genes, such as those coding for chemokine receptors, *CCR6* and *CXCR3*, we observed a general reduction in the induction of their expression following activation. Therefore, we can assume that RIZ2 strengthens the polarization of T lymphocytes in Th2. RIZ1, as RIZ2, is capable of repressing *CCR6* and *CXCR3* but not of increasing *CCR4* levels. Probably the effect could be due to the concomitant repression of *BLIMP1b* (Fig. 7).

Interestingly, the *CD40LG* high expression level in basal condition, which was particularly observed in RIZ1 overexpressing cells than RIZ2 ones, and the *CD40LG* up-regulation, observed during cell activation, could be related to the variations in the expression levels of positive and negative regulators induced by the adaptation of the cell line to the overexpression of PRDM2 forms that mimic key steps of cell activation or memory cell differentiation.

In summary, the stable forced expression of RIZ1 or RIZ2 in clones of Jurkat T cell line induced a significant variation in the expression level of key transcription factors involved in T lymphocyte differentiation observed already in steady state. Accordingly, these clones showed a different ability to modulate the expression level of these transcription factors upon cell activation. The *PRDM1a/b* balance has shifted in favor of *BLIMP1a* in RIZ1-overexpressing cells and in favor of *BLIMP1b* in RIZ2-overexpressing cells. In addition, *GATA3* expression level is noticeably modified by both RIZ1 and RIZ2.

## Conclusions

Overall, our observations represent the first step for the characterization of *PRDM2* in lymphocyte activation/differentiation. In the future, we point out to clarify deeply the mechanism of action and functions of *PRDM2* in lymphocyte activation/differentiation. In our opinion, it could be interesting evaluating the gene expression regulation by performing time-course Next Generation Sequencing (NGS) analysis in single cell. Furthermore, it could be remarkable to study the chromatin architecture of genes regulated by PRDM2 proteins, identifying any PRDM2 responsive element sequence. Considering that literature data shows the involvement of other genes of the family, including *PRDM15* in lymphoproliferative diseases [60], the preliminary evidence of our study allows us to hypothesize the use of drugs directed against RIZ or BLIMP1 to prevent or modify the response of cells to activation and cell differentiation and as a useful tool also for cancer therapy.

Consistent with its established role as an essential regulator of immune cell function, numerous *PRDM1-*associated risk alleles have been linked with autoimmune pathologies [15, 68]. Noteworthy, a recent meta-analysis of multi-trait genome-wide association studies identified *PRDM2* as a locus associated with six autoimmune and allergic diseases thus suggesting the implication of this gene [69]. Abnormal gene expression level or expression of genes containing deleterious variants can be at the basis of these genetic diseases. Currently, several molecular tools have been developed and are in clinical trials for therapeutic purposes acting to restore the physiological gene expression [70]. Thus, the whole knowledge of the transcription regulation mechanisms involving *PRDM1* and *PRDM2* genes in the immune biology can provide the molecular bases for the application of these new strategies to the autoimmune diseases.

## Supplementary Information

**Additional file 1**: **Figure S1**: Analysis of T cell subpopulations and activated cells at 48h by flow cytometer. CD4+, CD8+ T lymphocytes, and T naïve cells were analyzed by 6 Color TBNK + Truc assay with BD FACSLyricTM flow cytometer. The percentage of activated cells (scatter plots in blue) was measured at 48h with BD MultitestTM CD8/CD38/CD3/HLA-DR and anti-CD4 PECy7 and anti-CD45 V500C. **Figure S2**: Lymphocyte activation increases *PRDM1 *and *PRDM2 *expression levels. RT-PCR analysis of *PRDM1 *and *PRDM2 *transcripts upon activation in naïve T cells after stimulation with anti-CD3/CD28 and PMA/Ion for 2h and 6h. Semiquantitative analysis of genes related to lymphocyte activation is also reported. Bar graphs represent quantitative data obtained by ImageJ analysis of gel electrophoresis images. **Figure S3**: **A. **Schematic presentation of primer localizations for selectively measuring the expression levels of transcript variants with different 3' ends generated by alternative splicing [10]. The use of specific reverse oligonucleotides allowed to distinguish two amplicons with different tails. **B. **Bar graphs represent data from qRT-PCR analysis of *RIZ* *ex10 *amplicon ad *RIZ ex9a *amplicon at different time points. Expression levels were calculated using the DDCt method with the indicated control gene. The ratio between the *RIZ ex10 *and *RIZ ex9a *amplicons was also calculated. Three independent experiments in triplicates were performed and data expressed as mean ± SD. ns (not significant), *P<0.05 *vs *control cells. *p<0.05, **p<0.01, ***p<0.001. **Figure S4**: *PRDM2 *transcript levels in Jurkat cell line. Expression levels of *RIZ1 *and *RIZ2 *and their proteins in Jurkat cells after transfection with the indicated plasmids through qRT-PCR and Western blot analyses respectively.

## Data Availability

The datasets used and/or analysed during the current study are available from the corresponding author on reasonable request.

## Abbreviations

AKT: Protein kinase B
APC: Antigen presenting cell
BACH2: BTB Domain and CNC Homolog 2
BCL6: B cell lymphoma 6
BLIMP1: B lymphocyte-induced maturation protein 1
CCR C–C: Motif Chemokine Receptor
CD40LG: CD40 Ligand
CIITA MHC: Class II trans-activator
CML: Chronic myelogenous leukemia
CTLA-4: Cytotoxic T-Lymphocyte Antigen 4
CXCR: C-X-C Motif Chemokine Receptor
DLBCL: Diffuse Large B-Cell Lymphoma
FBS: Fetal bovine serum
FOXP3: Forkhead box P3
GAPDH: Glyceraldehydes-3-phophate dehydrogenase
HMTase: Histone methyltransferases
IFN: Interferon
IL: Interleukin
IL2RA: Interleukin 2 receptor subunit alpha
Ion: Ionomycin A
IRF: Interferon regulatory factor
IRS: Insulin receptor substrate
KLF2: KLF Transcription Factor 2
MAPK: Mitogen-activated protein kinase
MEK: Mitogen-activated protein kinase kinase)
PAX5: Paired box 5
PBMCs: SPeripheral blood mononuclear cells
PI3K: Phosphatidylinositol 3-kinase
PMA: Phorbol 12-myristate 13-acetate
PRDMs: PRDF1 and RIZ1 homology domain containing proteins
RIZ: Retinoblastoma protein interacting zinc finger gene
RORC: RAR Related Orphan Receptor C
RPS18: Ribosomal protein S18
STAT: Signal transducer and activator of transcription
TBX21: T-Box Transcription Factor 21
TCR: T cell receptor
Th: T helper
TNFR: Tumor necrosis factor receptor
