# Supplementary material for: Exploring the putative role of PRDM1 and PRDM2 transcripts as mediators of T lymphocyte activation
Source: J Transl Med. 2023 Mar 24;21:217. doi: 10.1186/s12967-023-04066-x (PMC10039509; doi:10.1186/s12967-023-04066-x)
Supplement: Supplementary file 1 — Additional file 1: Figure S1: Analysis of T cell subpopulations and activated cells at 48h by flow cytometer. CD4+, CD8+ T lymphocytes, and T naïve cells were analyzed by 6 Color TBNK + Truc assay with BD FACSLyricTM flow cytometer. The percentage of activated cells (scatter plots in blue) was measured at 48h with BD MultitestTM CD8/CD38/CD3/HLA-DR and anti-CD4 PECy7 and anti-CD45 V500C. Figure S2: Lymphocyte activation increases PRDM1 and PRDM2 expression levels. RT-PCR analysis of PRDM1 and PRDM2 transcripts upon activation in naïve T cells after stimulation with anti-CD3/CD28 and PMA/Ion for 2h and 6h. Semiquantitative analysis of genes related to lymphocyte activation is also reported. Bar graphs represent quantitative data obtained by ImageJ analysis of gel electrophoresis images. Figure S3: A. Schematic presentation of primer localizations for selectively measuring the expression levels of transcript variants with different 3' ends generated by alternative splicing [10]. The use of specific reverse oligonucleotides allowed to distinguish two amplicons with different tails. B. Bar graphs represent data from qRT-PCR analysis of RIZ ex10 amplicon ad RIZ ex9a amplicon at different time points. Expression levels were calculated using the DDCt method with the indicated control gene. The ratio between the RIZ ex10 and RIZ ex9a amplicons was also calculated. Three independent experiments in triplicates were performed and data expressed as mean ± SD. ns (not significant), *P<0.05 vs control cells. *p<0.05, **p<0.01, ***p<0.001. Figure S4: PRDM2 transcript levels in Jurkat cell line. Expression levels of RIZ1 and RIZ2 and their proteins in Jurkat cells after transfection with the indicated plasmids through qRT-PCR and Western blot analyses respectively. [file 12967_2023_4066_MOESM1_ESM.pdf]

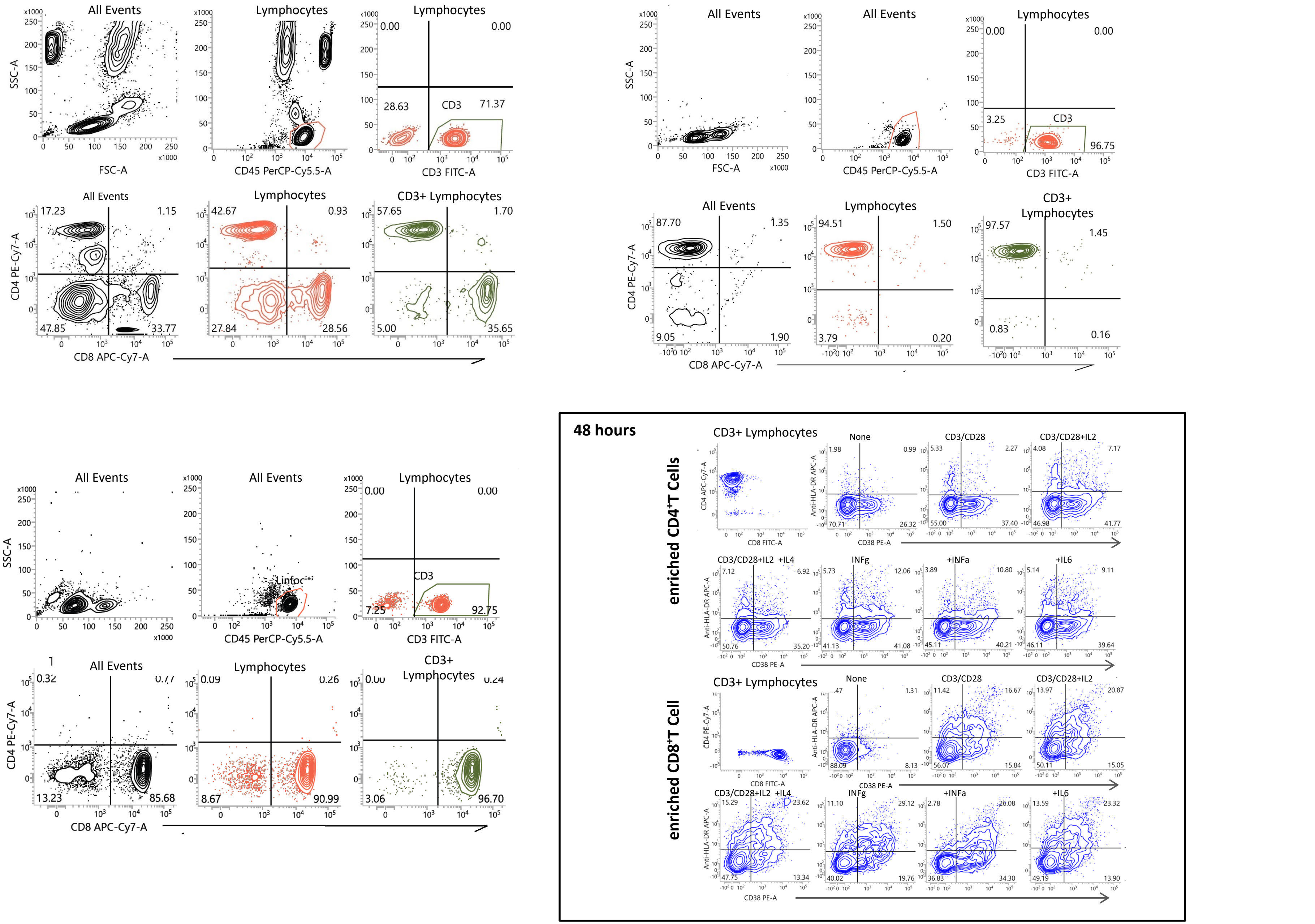

Figure S1: Analysis of T cell subpopulations and activated cells at 48h by flow cytometer. $CD4^+$, $CD8^+$ T lymphocytes, and T naïve cells were analyzed by 6 Color TBNK + Truc assay with BD FACSLyric^{TM} flow cytometer. The percentage of activated cells (scatter plots in blue) was measured at 48h with BD Multitest^{TM} CD8/CD38/CD3/HLA-DR and anti-CD4 PECy7 and anti-CD45 V500C.

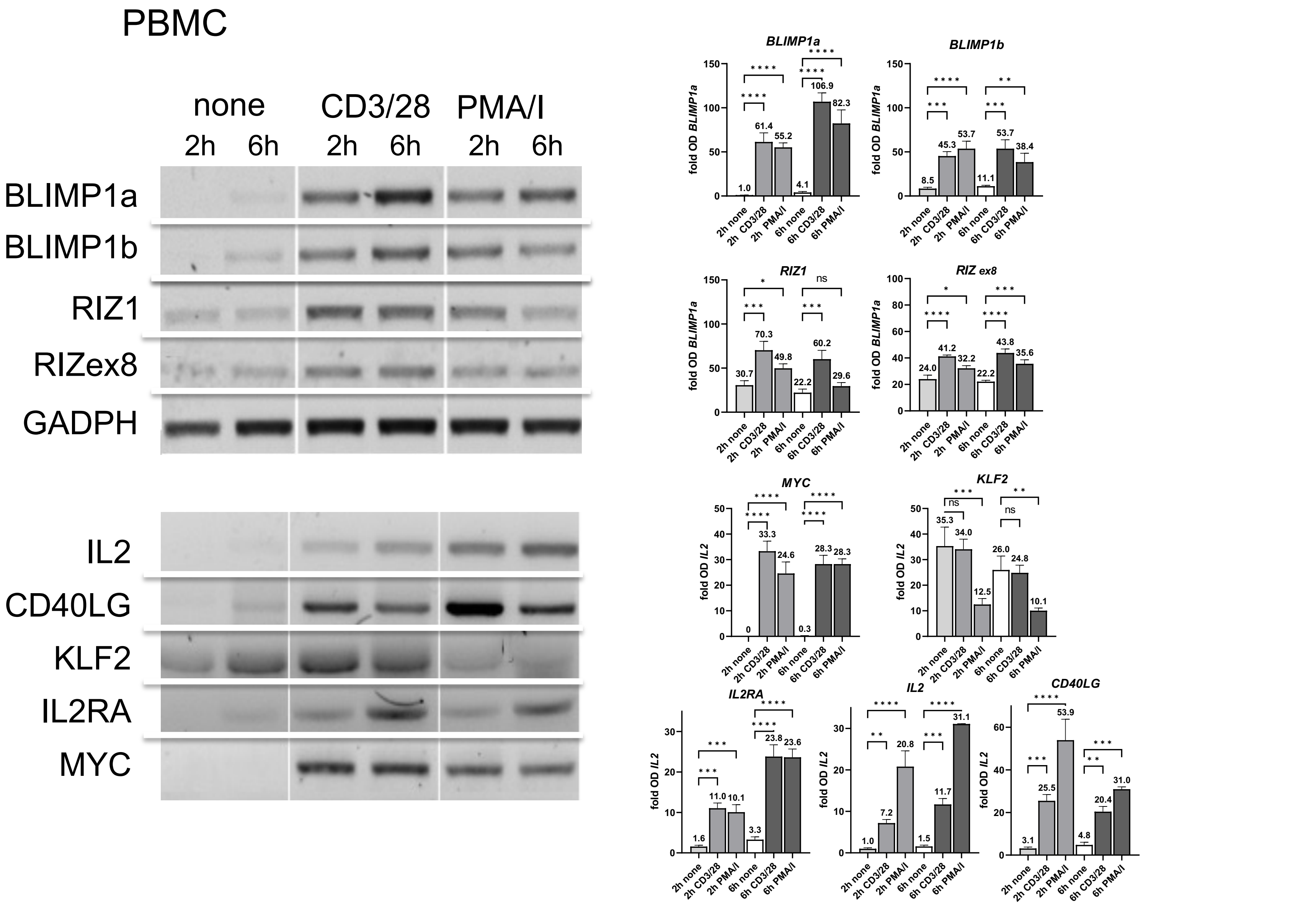

Figure S2: Lymphocyte activation increases *PRDM1* and *PRDM2* expression levels. RT-PCR analysis of *PRDM1* and *PRDM2* transcripts upon activation in naïve T cells after stimulation with anti-CD3/CD28 and PMA/Ion for 2h and 6h. Semiquantitative analysis of genes related to lymphocyte activation is also reported. Bar graphs represent quantitative data obtained by ImageJ analysis of gel electrophoresis images.

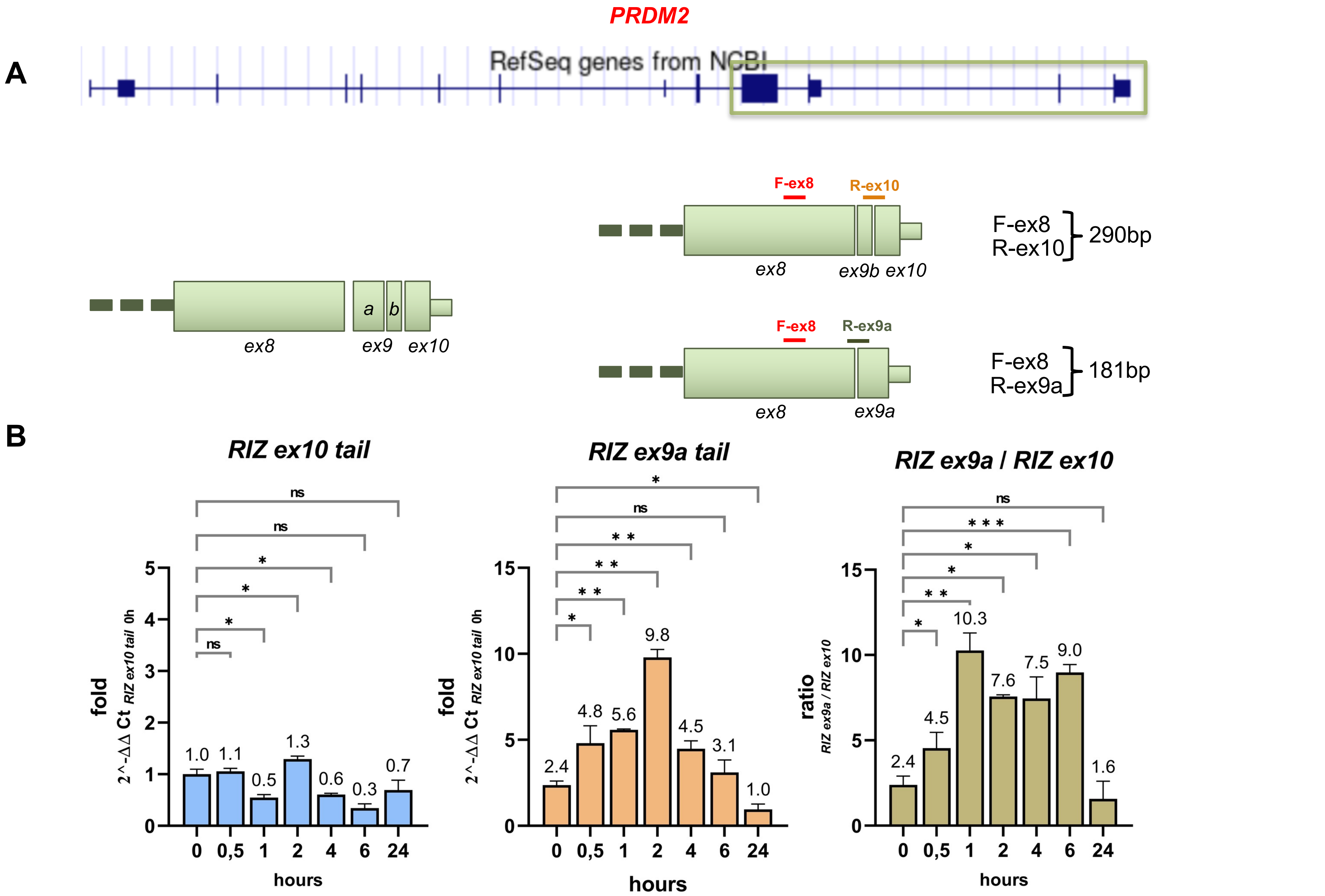

Figure S3: **A.** Schematic presentation of primer localizations for selectively measuring the expression levels of transcript variants with different 3' ends generated by alternative splicing [10]. The use of specific reverse oligonucleotides allowed to distinguish two amplicons with different tails. **B.** Bar graphs represent data from qRT-PCR analysis of *RIZ ex10* amplicon ad *RIZ ex9a* amplicon at different time points. Expression levels were calculated using the ΔΔCt method with the indicated control gene. The ratio between the *RIZ ex10* and *RIZ ex9a* amplicons was also calculated. Three independent experiments in triplicates were performed and data expressed as mean ± SD. ns (not significant), **P*<0.05 *vs* control cells. *p<0.05, **p<0.01, ***p<0.001.

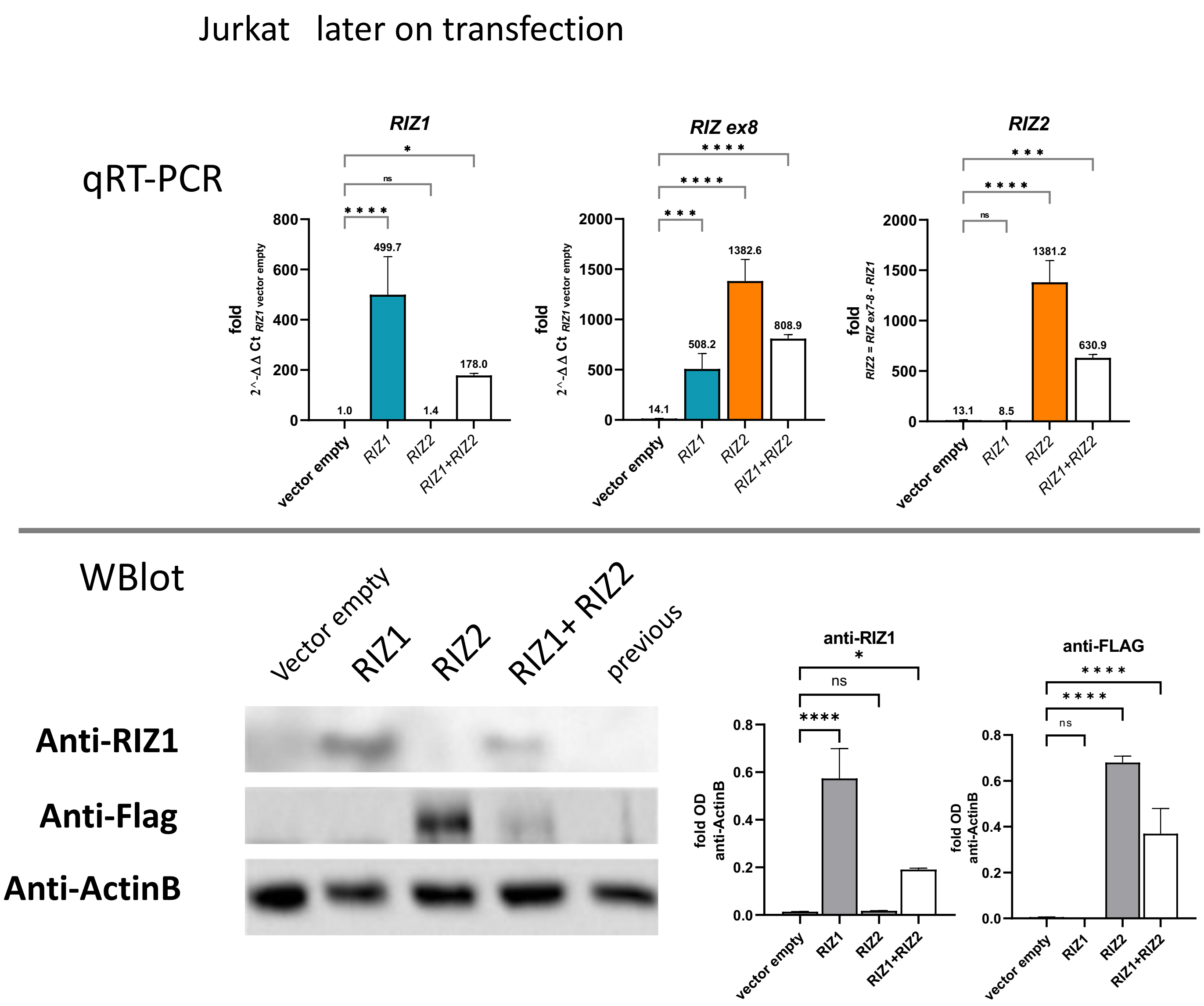

Figure S4: *PRDM2* transcript levels in Jurkat cell line. Expression levels of *RIZ1* and *RIZ2* and their proteins in Jurkat cells after transfection with the indicated plasmids through qRT-PCR and Western blot analyses respectively.
